# A Uterine Fibroid Operation1Read before the Wyoming County Medical Association, April 13, 1897.

**Published:** 1897-07

**Authors:** Wm. Stanton

**Affiliations:** Varysburg, N.Y.


### Clinical Report.

### A UTERINE FIBROID OPERATION.[1]

BY DR. M. D. MANN, Buffalo, N. Y.

REPORTED BY WM. STANTON, M. D., Varysburg, N. Y.

LATE Wednesday P. M., February 3d, I was called to see Mrs. T. I found her in bed, suffering from occasional pains which, she said, were "exactly like labor pains." She appeared well nourished, and had done her own housework until the day previous. Countenance was good, except a peculiar anxious expression. Temperature, 100°. Pulse, 90, and respiration, 22. She gave the following history: Age, 42. Mother of two children, aged 15 and 18 years. Had a miscarriage ten years ago. No other pregnancies. Menstruates regularly, but suffers from dysmenorrhea and leucorrhea. About three years ago she noticed something wrong in the vagina, at first only being aware of its presence at the menstrual period. After a time, however, there was a constant sensation, as of some object in the vagina. During the following two years she was treated by one or two irregulars for falling of the womb. One or two members of this Association who examined her gave her no treatment, but urged her to consult a specialist.

Meanwhile the growth had steadily increased in size until it filled the vagina, and at times would protrude through the vulva; then she would put it back by lying down and manipulating it much the same as a hernia.

For about a year she had consulted no one and taken no treatment. Some four weeks previous she had slipped on an icy step and slid down in a sitting position, striking heavily on five or six steps, but had noticed no ill effects beyond the momentary jar. The day before, she began to suffer with severe pains, resembling labor pains, but it being near the expected menstruation and she accustomed to similar pains at this time, thought nothing of it until the tumor was expelled from the vulva. She still believed it to be her uterus enlarged and deceased, and, taking her

1. Read before the Wyoming County Medical Association, April 13, 1897.

bed, attempted to replace it. Failing in this, she called me to perform the service for her.

On examination I found protruding from the vulva a rather soft, yielding tumor, the size of a seven-months' fetal head, which was covered by a membrane resembling mucus and of a dark, purplish color, suggesting a large placenta in its membranes. It was attached by a pedicle about two inches in diameter and four inches long, as stretched. Following up this pedicle it seemed to spread out again into a solid mass of tissue, which completely filled the pelvic cavity. I could not find the cervix or any trace of it, although when she had pains a solid body, about the size of a normal uterus, could be plainly felt in the abdomen, like a nodule on the mass in the pelvis. I diagnosticated uterine fibroid and advised its removal as soon as possible. Counsel being desired, Dr. Seeley was called and he concurring in diagnosis and advice Dr. Mann was called.

Thursday she began to show unmistakable symptoms of septicemia, and as the exposed growth was showing signs of disintegration I ligated the pedicle as high as possible and removed the tumor. The pains ceased and did not return until Friday noon, but the septic symptoms steadily advanced. About noon Friday she began to have terrific pains and in a short time gave birth to a second tumor, resembling the first in all respects except size. This one was fully as large as a man's head. It was now plain how the first pedicle spread out above. This growth had so completely blocked the vagina that we failed to find any passage by it. There was a quantity of pus retained above it and its attachment was to the cervix by a short pedicle, about two and one-half inches in diameter. The os was now easily found and, like the entire uterus, was almost normal. Very soon after expelling this she began to suffer from profound shock and only by the hypodermic use of strychnine, nitroglycerine and quantities of brandy, together with slings, hot applications, rubbing, enemas of whisky, and the like, did we keep her alive until Dr. Mann came at 5.15 P. M.

She was hastily prepared for operation, not with any expectation of saving her, but as a last and only resort. She took ether finely, her pulse improved under it, she came quickly out of it, and at 5.45 was back in bed. She seemed to rally for a time, was conscious and rational. Pulse better than for thirty-six hours, but then she began to fail and went down in spite of our best efforts. She died one hour after the operation, overcome by the septic poisoning.

The larger tumor weighed 4½ lbs. The smaller one I estimated at 1½ lbs. They had apparently been of loose, fibrous structure and quite vascular. Decomposition was far advanced and the large pedicle had allowed the products of decomposition to pass freely into the circulation. It was thought perhaps the violence of the expelling pains had torn the peritoneum, or forced some of the retained pus into the peritoneal cavity, but, a post-mortem being refused, we could neither verify nor disprove these suggestions.

The operation consisted in drawing down the uterus, removing the pedicle, clamping the bleeding surfaces, irrigating with hot sublimated solution and packing with iodofom gauze, with the clamps in position and a catheter in the bladder. Hemorrhage was slight and the operation very quickly performed, the patient not being under the anesthetic to exceed twelve or fifteen minutes, and was back in bed thirty minutes after Dr. Mann arrived.

---

Abuse of the Dispensary.—It may be broadly stated, as the result of exhaustive statistical study, that fully 50 per cent. of the patients who apply for free medical aid are totally undeserving of such charity. The main reason for this is that no effectual means are taken by the managers of these institutions to correct the abuse. For the sake of donations and the ostensible good accomplished by the treatment of a large number of patients, these charities are managed on the usual business principles of proving their right to be and to prosper on the assumed basis of demand and supply. In New York alone there are 116 dispensaries, each one of which is vying with the other in propagating the worst form of pauperism. The public is being taught that nothing is more freely given than medical advice to any who may ask for it. The institutions in question are crowded daily by hundreds of well-to-do patients, who are encouraged to defraud the really poor and to cheat the charitably disposed doctor of his legitimate fee. All this goes on in spite of protests and in open defiance of all the laws of ordinary decency and fair play. The managers of these socalled charities, who virtually have the matter in their own hands, while pretending to deplore present conditions are covertly combating every effort at reform on the ground of its impracticability.—Dr. George F. Shrady in June *Forum.*

---

Tuberculosis in Infants.—It would seem from recent inquiry that intrauterine infection seldom happens, even when the mother is suffering with tubercular disease in an advanced form. In nearly all infants dying from tuberculosis the germ entered the system through the air-passages.—*Western Medical Review.*

